# Supplementary material for: Learning curve and surgical outcome of robotic assisted colorectal surgery with ERAS program
Source: Sci Rep. 2022 Nov 29;12:20566. doi: 10.1038/s41598-022-24665-w (PMC9709162; doi:10.1038/s41598-022-24665-w)
Supplement: Supplementary file 2 — Supplementary Information 2. [file 41598_2022_24665_MOESM2_ESM.zip › RAL Raw data-Ñ[▒K/129/HS_ñw╝╨░O▒Kñσ.pdf]

正式摘要(電子簽章：000000231068934)		
W52 / W52 ☐ 英文標題(注意:無英文姓名-請補病人基本檔!)	☐【讀取】憑證	

臺中榮民總醫院

出院病歷摘要

轉入號 : 轉出號 :

[redacted]

(8)流水編號	
(9)入院日期	110/11/23 (2021) 直腸外科 W52 - 162
(10)轉科日期	
(11)出院日期	110/11/30 (2021) 住院天數計 7 日

診斷

- 入院 :1. Rectal adenocarcinoma, 10cm FAV
  - s/p Capecitabine (2021/11/03-24)
  - s/p short course R/T, 25Gy/5fr. (2021/11/16-22)
  2. HTN, under medication
  3. Ureter stone, s/p ESWL and cystoscopy in 2011 and 2016 at Li-shin H.
- 出院 :1. Rectal adenocarcinoma, 10cm FAV
  - s/p Capecitabine (2021/11/03-24)
  - s/p short course R/T, 25Gy/5fr. (2021/11/16-22)
  - s/p Robotic LAR + loop ileostomy on 2021/11/24
  2. HTN, under medication
  3. Ureter stone, s/p ESWL and cystoscopy in 2011 and 2016 at Li-shin H.

主訴

- Small calibor stool for 3 months

病史

- This is a 44 years old male patient with history of

  1. HTN, under medication since 2020

  2. Ureter stone, s/p ESWL and cystoscopy in 2011 and 2016 at Li-shin H.

  According to the patient, he had small caliber stool for 3 months, and he went to other hospital for help, where Digital revealed finger tip palpable mass. Colonoscopy was arraged and showed 10cm FAV tumor. Further image survey such as CT was done and showed Rectal cancer and liver lesion, R/I metastasis. Thus, MRI and PET were done, and had no significant findings over liver. Then, he came to our CRS OPD for second opinion. After discussion, he received short course CCRT first. This time, he was admitted to our ward for surgical intervention.

體檢發現

-
  - General appearance :
    - easy looking , conscious clear , without cardiopulmonary distress
    - Vital signs自動帶入date 體溫(BT) 脈搏(P) 呼吸(R) 血壓(BP) 身高(BH) 體重(BW) BMI
      20211123 36℃ 88次/分 18次/分 136/91mmHg 172.3cm 70kg 23.6
    - Vital signs: B.P. 136/19 mmHg , P.R. 88 /min , R.R. 18 /min , B.T. 36 ℃
  - HEENT :
    - Eyes: no anemic conjunctiva, no icteric sclera, normal EOM
    - ENT: no otorrhea, no rhinorrhea, no erythematous mucosa, no injected tonsil, no deformity
  - Neck: supple, freely movable, trachea midline,no deformity, no engorged jugular vein , no palpable thyroid or lymph node enlargement
  - Chest:
    - Inspection: Grossly normal, symmetric and fully expansion, no accessory muscle used, no operation scar, no spider angiomata, no gynecomastia
    - Palpation: no palpable mass or nodule, no subcutaneous emphysema, no tenderness

-
  - Ausculation: clear breathing sound
- Heart:
  - Palpation: no thrill, no heave
  - Percussion: normal heart size
  - Ausculation: regular heart beat, normal S1, S2, no gallop, no murmur
- Extremities: No deformity, full ranged of motion without limitation, no clubbing finger or nail cyanosis, no pitting edema, no ulcer or pigmentation, no obvious skin lesion, no varicose vein, no bone, joint or muscle tenderness

CRS CONDITION

- Karnofsky performance status: (100) Normal, no complaints, no evidence of disease
- PE of Abdomen:
- CEA: 13.6 ng/ml
- Imaging Studies:
- Colonoscope report: .
- Figures:
  1. colon & rectum

手術日期及方法

- 手術日期及方法20211124 1. Robotic (XI) assisted low anterior resection + loop ileostomy
  2. Intraoperative colonoscopy : (-).
  3. High ligation (+) : At IMA root
  4. Take down from the splenic flexure : (-)
  5. Robotic EndoGIA (45-4.3) X2 + EEA (29mm) were applied to complete the anastomosis
  6. Proximal cut end: > 10 cm, distal cut end: 1.5 cm
  7. No drainage tube was inserted
  8. ADHESION BARRIER FILM was placed
  9. Loop ileostomy (About 15~20cm from IC valve) was done over RLQ of abdomen (Proximal limb : Ventral site)

住院治療經過

- After admission, he received Robotic LAR+loop ileostomy on 2021/11/24. Despite under ERAS protocol, we delyed removal of Foley catheter due to wound pain. Vomiting had been noticed, so we kept him liquid diet. Foley catheter was removed on 11/28. The abdominal fullness improved after removal of ileostomy rod. He had better appetite gradually. Under relative stable condition, he was discharged on 2021/11/30.

合併症

一般檢查

- 累積報告 :

DATE	TIME	ALB	TP	BIL.T	BIL.D	ALKP	AST	ALT	LDH	AMY	LIPASE	NH3	Ferritin	UA
1101124	1202		6.5											4.9
1101124	2120	3.9		1.90				33						
1101129	0737			1.61	0.49	74	18	33						

DATE	TIME	WBC	RBC	HGB	HCT	MCV	PLT	BLST	NEUT	BAND	SEG	LYM	MON	EOS	BAS
1101124	2120	10300		13.4	40.1		240		93.6			2.7	3.6	0.0	0.1
1101129	0719	5260		14.0	42.0		190			1.0	74.0	10.0	15.0	0.0	0.0

DATE	TIME	NA	K	CL	BUN	CREAT	eGFR	CA	Mg	P	CRP	ESR	PCT	D-Dimer	LACTATE
1101124	2120	142	3.8		10	0.81		8.3							
1101129	0721														6.9
1101129	0737	133	3.6		16	0.74	122.12								

DATE	TIME	GLUCOSE	HbA1C	CHOL	TG	HDL	LDL	KETONE	CK	CKMB	TRO-T	PT-P	APTT-P	NT-pro BNP
1101124	1202					59	85							

- 放射線報告 :檢驗項目 **:CHEST AP VIEW (PORTABLE)** 報告日期 **:1101126**

  主　訴：
  Result:N
  Finding:
  > Cardiomegaly.
  > Mild increased interstitial infiltration in bilateral lungs.
  > Left costophrenic angle blunting, pleural effusion or pleural change could be considered.
  > On EKG leads, central venous catheter
  > The visible bony structure appears intact.
  Impression:
  As above description.

特殊檢查

病理報告

其它

出院時情況

- 3.改門診治療

出院指示

-

藥名	劑量	劑量單位	用法	頻次	數量	數量單位	現況	天數	單位
(口)Takepron OD tab 30mg	1	TAB	PO	QDAC	7	TAB	TH	7	天
Ultracet tab	1	TAB	PO	Q6H	28	TAB	TH	7	天

出院計劃

- 門診日期
  - date on 2021/12/07

主治醫師 2062F 蔣鋒帆 / 住院醫師 2339C 蔡濬樟

正式摘要**(電子簽章：000000231068934)**

W52 / W52

doctorRank:B
